# Aerial Prefeeding Followed by Ground Based Toxic Baiting for More Efficient and Acceptable Poisoning of Invasive Small Mammalian Pests

**DOI:** 10.1371/journal.pone.0134032

**Published:** 2015-07-28

**Authors:** David Morgan, Bruce Warburton, Graham Nugent

**Affiliations:** Department of Wildlife Ecology & Management, Landcare Research, PO Box 40, Lincoln 7640, New Zealand; University of Queensland, AUSTRALIA

## Abstract

Introduced brushtail possums (*Trichosurus vulpecula*) and rat species (*Rattus* spp.) are major vertebrate pests in New Zealand, with impacts on conservation and agriculture being managed largely through poisoning operations. Aerial distribution of baits containing sodium fluoroacetate (1080) has been refined to maximise cost effectiveness and minimise environmental impact, but this method is strongly opposed by some as it is perceived as being indiscriminate. Although ground based control enables precise placement of baits, operations are often more than twice as costly as aerial control, mainly due to the high labour costs. We investigated a new approach to ground based control that combined aerial distribution of non-toxic ‘prefeed’ baits followed by sparse distribution of toxic baits at regular intervals along the GPS tracked prefeeding flight paths. This approach was tested in two field trials in which both 1080 baits and cholecalciferol baits were used in separate areas. Effectiveness of the approach, assessed primarily using ‘chewcards’, was compared with that of scheduled aerial 1080 operations that were conducted in outlying areas of both trials. Contractors carrying out ground based control were able to follow the GPS tracks of aerial prefeeding flight lines very accurately, and with 1080 baits achieved very high levels of kill of possums and rats similar to those achieved by aerial 1080 baiting. Cholecalciferol was less effective in the first trial, but by doubling the amount of cholecalciferol bait used in the second trial, few possums or rats survived. By measuring the time taken to complete ground baiting from GPS tracks, we predicted that the method (using 1080 baits) would be similarly cost effective to aerial 1080 operations for controlling possums and rats, and considerably less expensive than typical current costs of ground based control. The main limitations to the use of the method will be access to, and size of, the operational site, along with topography and vegetation density.

## Introduction

Invasive small mammal pests pose major threats to conservation and production values globally, but are typically expensive to control or eliminate at large scales. In New Zealand, two major pests, the Australian brushtail possum (*Trichosurus vulpecula*) and the ship or black rat (*Rattus rattus*) are controlled by poisoning over millions of hectares. The possum is a nocturnal, arboreal, largely herbivorous fur-bearing marsupial about the same size as the domestic cat. It was introduced from Australia to New Zealand over 160 years ago to establish a fur trade, and has become a major pest, causing extensive damage to conservation values, agricultural crops and forest plantations, and by spreading bovine tuberculosis [1]. A widely used method of control for the last 50 years has been poisoning using sodium fluoroacetate (i.e. ‘1080’) baits [2]. Baits are distributed aerially by GPS guided aircraft (i.e. ‘aerial baiting’), or in ground based operations by licensed contractors (i.e. ‘ground baiting’). Such operations are often also designed to target the three species of introduced rats, particularly *R*. *rattus* which also has major impacts on native fauna [3].

Aerial 1080 bait has become the preferred option in remote, steep or heavily forested and scrubby landscapes where ground access and travel are difficult and time consuming. Baits are sown along parallel flight paths spaced 100–280m apart, using either broadcasting sowing in an effort to spread bait evenly over the whole area, or strip or cluster sowing in which bait is concentrated in narrow swaths a few tens-of metres wide under the flight path [4]. Typically, aerial baiting involves an initial sowing of non-toxic baits (i.e. ‘prefeeding’) followed by a later sowing of toxic bait. Prefeeding serves to familiarise possums with the bait in order to increase the likelihood that they will consume a lethal dose on first encounter with toxic bait [5]. When bait (prefeed and toxic) is distributed in strips or clusters it is also likely to aggregate possums along the centre of flight paths where they are most likely to subsequently encounter toxic baits [6], which are generally applied 5–10 days after prefeeding. While the technique has been refined to maximise cost effectiveness and minimise the amount of 1080 being distributed in the environment [7,8], aerial distribution of 1080 is strongly opposed by some New Zealanders, partly because of opposition to 1080 itself and partly because of opposition to aerial delivery, as it is perceived as being an uncontrolled and indiscriminate technique for placing bait [9]. Use of other toxins would overcome 1080-specific opposition, but none are registered for aerial control of possums (and registration of a new aerial toxin would be difficult). Ground baiting overcomes the concerns over aerial delivery, but the combination of low travel speeds, small payloads and (proportionately) higher labour costs can make ground baiting expensive in country that difficult to access.

We therefore explored the feasibility and efficacy of a new approach to ground baiting that combines aerial baiting for the prefeeding phase of the operation followed by ground baiting to deliver comparatively small amounts of toxic bait (using either 1080 or an alternative toxin) along the prefeed flight paths. Prefeeding by aerial delivery offers greater efficiency [4,6,10], while toxic ground baiting avoids the much more complex and costly planning and consent requirements for aerial application of toxic baits [11,12].

Our approach is based on findings from recent research into refining aerial baiting strategies: this showed that both aerial and ground based sowing of bait in highly aggregated strips or clusters at rates of 0.17–0.5 kg/ha of 1080 bait was usually as effective in reducing possum and rat densities as the current standard practice of distributing bait evenly using conventional ‘broadcast’ aerial sowing at rates of 1.5–2.0 kg/ha of 1080 bait [4,8,10]. The ability to achieve reductions with comparatively very low sowing rates makes ground baiting more feasible by greatly increasing the area that can be treated with a single load of bait. Even with the much reduced overall sowing rates, bait density within the clusters or strips is still higher than with broadcast dispersion [10]. Furthermore, the production of sub-lethal fragments of baits that can occur is greatly reduced by avoiding the impact forces from conventional sowing buckets concomitant with achieving broadcast baiting [13]. The restricted placement of prefeed does not impede rapid discovery by possums: a field study of the movements of 22 radio collared possums showed that most were immediately attracted from as far away as 250m to a line of prefeed (visits peaking on the second day of prefeed availability), and possums continued to look for bait for up to 18 days after all bait was removed [6]. We therefore aimed to emulate this approach by using ground laying of toxic bait in clusters along the flight paths used to aerially sow non-toxic prefeed. Our first aim was to determine how accurately and quickly contractors were able to lay toxic baits at specified intervals along the prefeed flight paths.

Our second aim was to assess the cost effectiveness of aerial prefeeding followed by ground baiting using an alternative toxin (cholecalciferol) in comparison with 1080. Cholecalciferol is registered for ground (but not aerial) control of possums and is often acceptable where the use of 1080 faces public opposition. Its advantages over 1080 include a lower risk of secondary poisoning, lower toxicity to birds, lower risks of residues in sub-lethally dosed game and livestock, and availability to users without the need for a licence [14]. However, because cholecalciferol baits are considerably more expensive than 1080 baits, it is imperative that the amount used is minimised without reducing operational effectiveness.

Our overarching aim was therefore to halve the long-run average annual cost of ground based possum control, by developing methods for aerially prefeeding treatment areas followed by ground baiting with either 1080 or cholecalciferol baits. To achieve that, we conducted two field trials to (i) determine if GPS receivers function adequately under a dense forest canopy to enable contractors to accurately follow aerial prefeeding flight paths, (ii) determine the rate of bait application achieved by ground control contractors, (iii) compare reductions in possum and rat populations achieved by conventional aerial non-toxic prefeed/aerial 1080 baiting with those achieved by aerial non-toxic prefeed/ground baiting (1080 and cholecalciferol), and (iv) compare the cost effectiveness of the three treatments.

## Methods

### Ethics statement

All studies were conducted in compliance with the New Zealand Animal Welfare Act (1999) under the approvals (numbers 08/09/02 and 11/03/02) and monitoring of the Landcare Research Animal Ethics Committee.

### Field trials

#### Study sites

**Fig 1:**
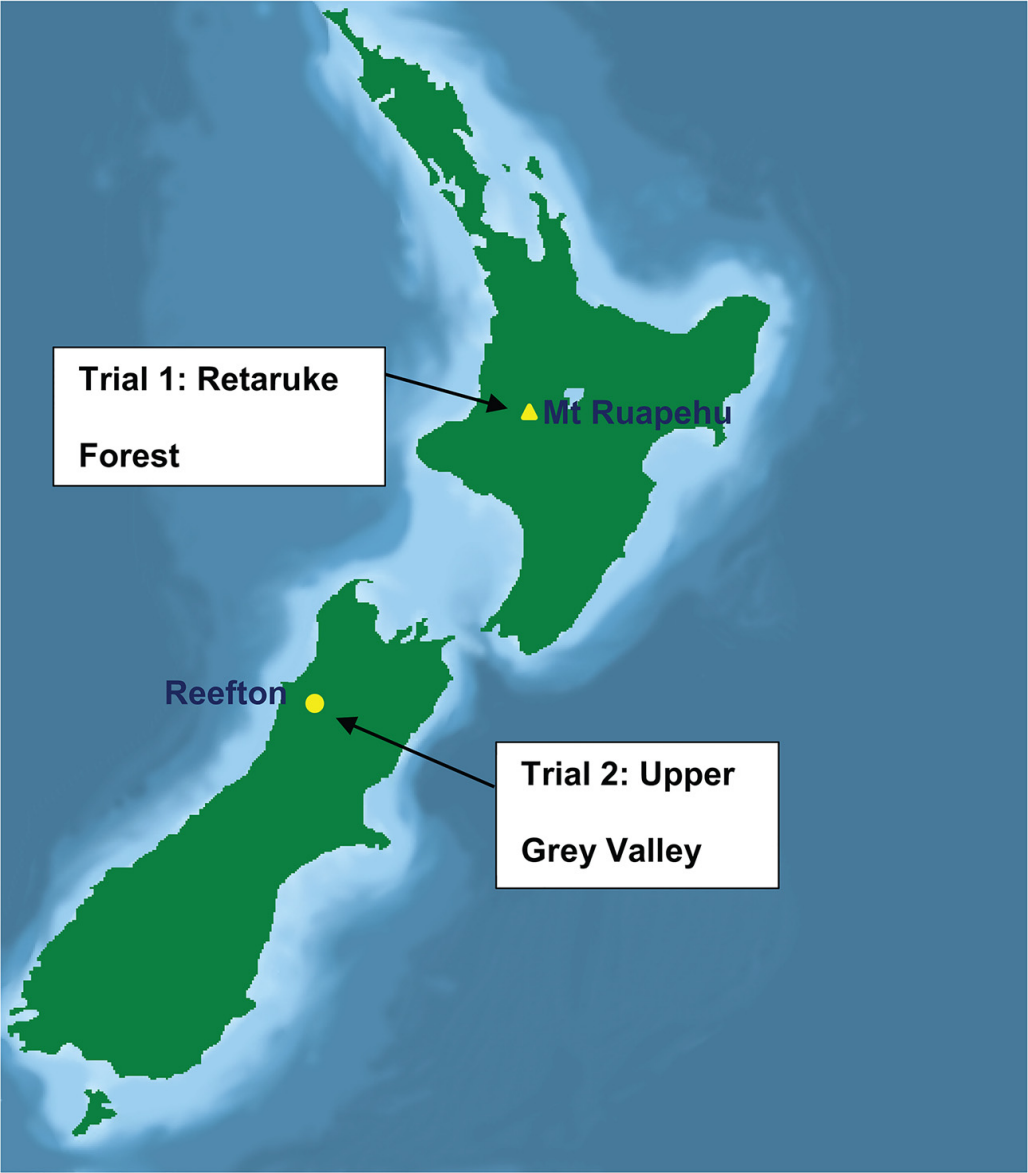
Location of the two trial sites in North and South Islands of New Zealand.

Two field trials were carried out in conjunction with aerial 1080 operations conducted by TBfree New Zealand (the agency responsible for managing bovine tuberculosis in New Zealand). Trial 1 was conducted during June-August 2012 in the Retaruke Forest, about 20 km due west of Mount Ruapehu (Fig 1). Landholder’s approval to conduct a trial at this site was granted by the New Zealand Department of Conservation (DOC; approval number DOCDM-1010140).The trial area was located in tall forest dominated by tawa (*Beilschmiedia tawa*) and kāmahi (*Weinmannia racemosa*) on gently undulating terrain at around 700m above sea level. Possum density was considered low to moderate, based on a 2011 operational assessment using the nationally standard Residual Trap Catch Index method (RTCI; [15]). This indicated that 12.3 ± 6.7% (95% CI) of leg-hold trap nights resulted in a possum capture ranging from 0 to 30% on individual lines (unpublished TBfree New Zealand data, 2011). The RTCI represents the mean number of possums captured per 100 trap nights (expressed as a percentage) after correcting for traps that are inactivated by capturing a non-target animal or being triggered but failing to catch an animal (half a ‘trap night’ was deducted from the total number of ‘available’ trap nights to account for these events, and comprised <1% of the total).

Trial 2 was conducted during July-September 2013 in the Upper Grey Valley, about 25km due south of Reefton (Fig 1), at an elevation of about 300m above sea level. Landholder’s approval was granted by DOC (approval number DOCDM-1251942). The trial was carried out in regenerating forest dominated by red beech (*Fuscospora fusca*) on mostly gently undulating ground, but with an area of steep gorge terrain deliberately included to assess the capability of contractors to carry out control to specifications in more difficult terrain. The possum population had not previously been controlled at this site and no previous population census data were available: however, a subjective assessment from a 2d site inspection suggested a low-moderate population density over the trial areas, similar to the Retaruke population before control.

#### Bait treatments Trial 1

Most of the 1210ha operational area was treated conventionally with aerially broadcast 1080 poison (preceded by aerially-broadcast prefeed baits), but two blocks of approximately 180ha each received aerial strip prefeeding before ground-laying of either commercially available 1080 ‘RS5’ baits (Animal Control Products, Wanganui, New Zealand) or experimental ‘Kolee’ (cholecalciferol) baits (Pest Control Research, Christchurch, New Zealand). Both bait types comprised milled cereals with added sweeteners and are formed into cylindrical pellets through an extrusion die. Treatments were as follows (and detailed in Table 1).

**Table 1:** Bait types & sizes and application rates used for the three treatments in the two trials.

Trial	Treatment block	Area (ha)	Prefeed bait type	Prefeed aerial application rate (area wide) (kg/ha)	Toxic bait type (with % wt:wt of toxin)	Toxic bait application rate (area wide) (kg/ha)
1	Aerial 1080	864	16mm RS5 non-toxic pellet	2.0	16mm RS5 pellet with 0.15% 1080	2.0
	Ground-laid 1080	174	10mm RS5 non-toxic pellet	1.0	20mm RS5 pellet with 0.15% 1080	0.5
	Ground-laid Kolee	181	10mm RS5 non-toxic pellet	1.0	20mm Kolee pellet with 0.8% cholecalciferol	0.5
2	Aerial 1080	22,426	16mm RS5 non-toxic pellet	2.0	20mm RS5 pellet with 0.15% 1080	2.0
	Ground-laid 1080	241	16mm RS5 non-toxic pellet	1.0	20mm RS5 pellet with 0.15% 1080	0.5
	Ground-laid Kolee	249	16mm RS5 non-toxic pellet	1.0	20mm Kolee pellet with 0.8% cholecalciferol	0.96

For aerial 1080 baiting, a single prefeed of 16mm (diameter) non-toxic bait was broadcast on 10 July 2012 with both the broadcast-baited swath width and the flight path spacing (FPS) being 180m. Toxic baiting was conducted 10d later, with broadcast sowing of 16mm baits containing 0.15% 1080 at 2 kg/ha, at the same 180m FPS but with no particular effort made to align the prefeed and toxin flight paths.

**Fig 2:**
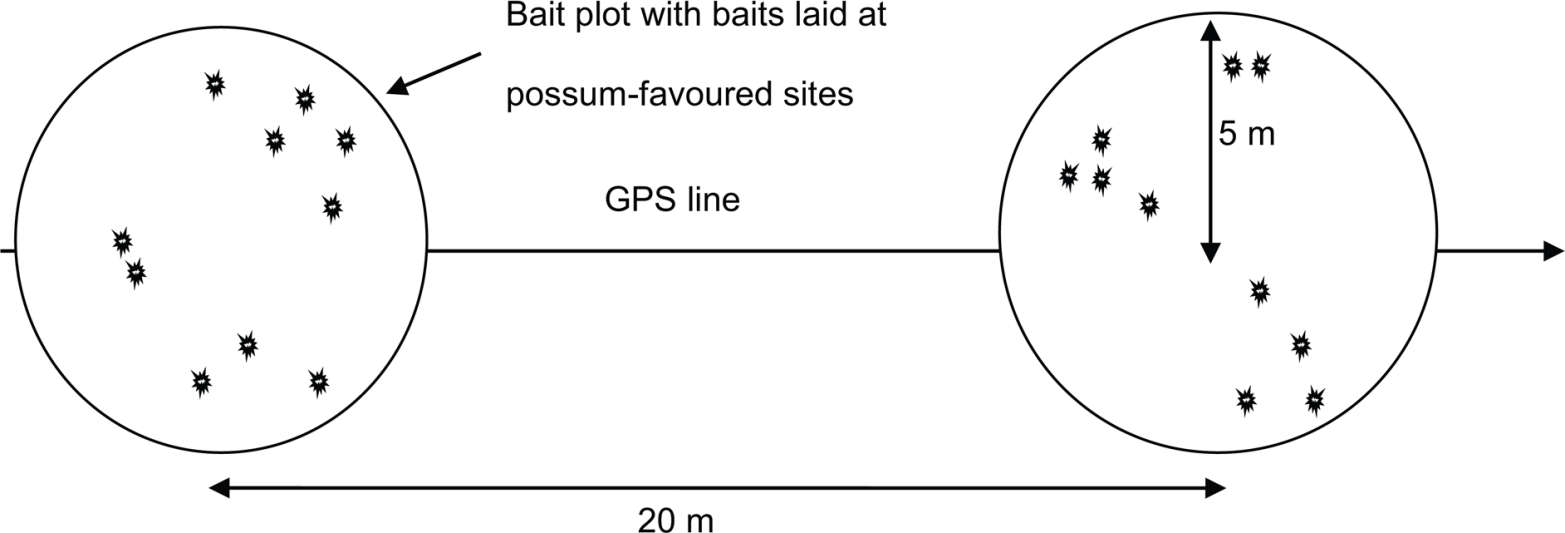
Diagram of the method of hand laying 1080 and Kolee used in both trials. Lines followed the GPS tracks of the aerial prefeed lines spaced 100m apart.

For ground-baiting with 1080, a single aerial prefeed of non-toxic 10mm baits was sown on 10 July 2012 at 1.7 kg per 100m of flight path with a baited swath width of 60m wide and a FPS of 100m. Pest control contractors (EcoFX, Otorohanga, New Zealand) carrying out ground-based control during 19–20 July (i.e. 9–10d after prefeeding) obtained the aerial prefeeding GPS ‘tracks’ (i.e. the positional data logged every 2 seconds during flight) from the aerial contractor, and were then required to log all ground-baiting points using Garmin GPS map 60CSx hand-held devices while following these tracks. Contractors laid about 100g of 20mm RS5 baits (i.e. 10 baits of mean weight c. 10g) containing 0.15% 1080 at sites favoured by possums (e.g. at the base of favoured tree species, or on flattened ‘runs’ made by repeated use by possums) in clusters of 5m radius spaced 20m apart along the prefeed flight paths (Fig 2). The smaller baits used during prefeeding were designed to increase the probability of bait encounter compared with larger baits sown at the same rate, but the larger baits were used for poisoning to reduce the risk of possums encountering sub-lethal baits. Laboratory assays showed the toxic baits, as also used in the aerial operation, contained 0.16% 1080, a variation of 7% above specification.

Ground baiting with cholecalciferol was undertaken as for ground-laid 1080 except the toxic bait used was 0.8% Kolee (cholecalciferol) 20mm cereal pellets. Laboratory assays showed the toxic baits contained 0.88% cholecalciferol, a variation of 13% above specification.

All baits (prefeed and toxic) contained 0.1% cinnamon (as a masking agent for any toxins present), and were surface-coated with a deer repellent (EPRO, Taupo, New Zealand) to minimise deer by-kill (as the area is popular for hunting) [16]. A Squirrel AS350FS2 helicopter (Wanganui Aeroworks, Wanganui, New Zealand) was used in all blocks for aerial bait delivery at a flying speed of 110 km/h. The sowing buckets used were calibrated by the aerial contractor to deliver specified sowing rates and swaths.

#### Bait treatments Trial 2

The second trial was carried out to the same specifications as the first, with three differences. First, larger (16mm) prefeed baits were used in all treatment areas because the smaller 10mm baits used in the first trial available were not available, but application rate was the same. Secondly, the application rate of Kolee baits was almost doubled to 0.96 kg/ha because results suggested that insufficient Kolee was presented in the first trial (see Discussion). Thirdly, there was no requirement to use EPRO deer repellent.

Laboratory assays showed that 1080 baits used in aerial and ground-laying contained 0.13% 1080 (i.e. 13% below specification) and Kolee baits contained 0.72% (10% below). Aerial prefeeding was completed during 27–29 July 2013, and ground-based toxic baiting on 9–18 August 2013, with an interval of 8–13d between prefeeding and toxic baiting in all areas except for a longer interval of 20–21d caused by prolonged bad weather affecting a small part of the aerial 1080 baiting area that was monitored.

### Data acquisition and analysis

**Fig 3:**
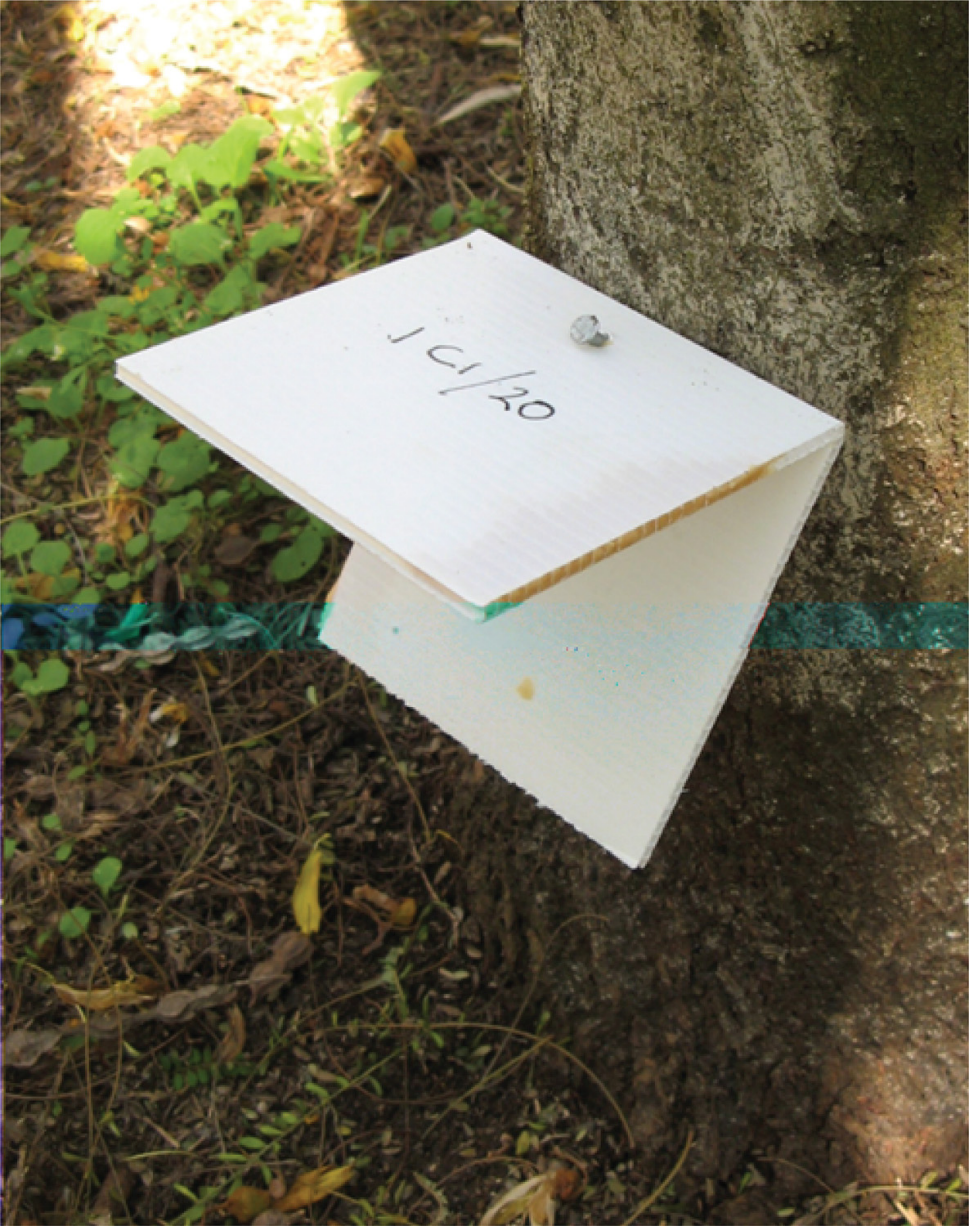
A ‘chewcard’ attached to a tree close to the ground. Peanut paste bait has been pressed into the hollow ribs of the card attracting interference by common pest species.

The GPS data from contractors were used to produce a visual display of the accuracy of navigation by importing the data into GIS and displaying them over an orthorectified satellite image (AIRBUS DS 2014, www.geo-airbusds.com/spotmaps/) and overlaying it on the aerial prefeeding GPS tracks obtained from the aerial operator. Images were downloaded and are here presented under license agreement between the New Zealand government (specifically – Crown Research Institutes) and Airbus Space & Defence Ltd (NZ Govt & non commercial EULA-SPOT6-7 v 08 05 2014). Actual time logged at the beginning and end of each baiting line enabled calculation of the mean time spent baiting plots and moving to the next plot, and the mean speed of bait laying. Comparisons of these parameters were made using analysis of variance (ANOVA) in the R statistical computing environment (version 2.15.1), with ‘trial’, ‘bait treatment’ and their interaction as factors. Mean values were compared using Tukey’s HSD tests.Effectiveness of baiting treatments was assessed by comparing the rates at which possums and rats were detected using ‘chewcards’[17] before and after toxic baiting (Fig 3). A simple index of interference is given by the percentage of cards interfered with (i.e. ‘CCI’—the Chewcard Index) before and after a specified period, which is varied depending on the estimated population density.

**Fig 4:**
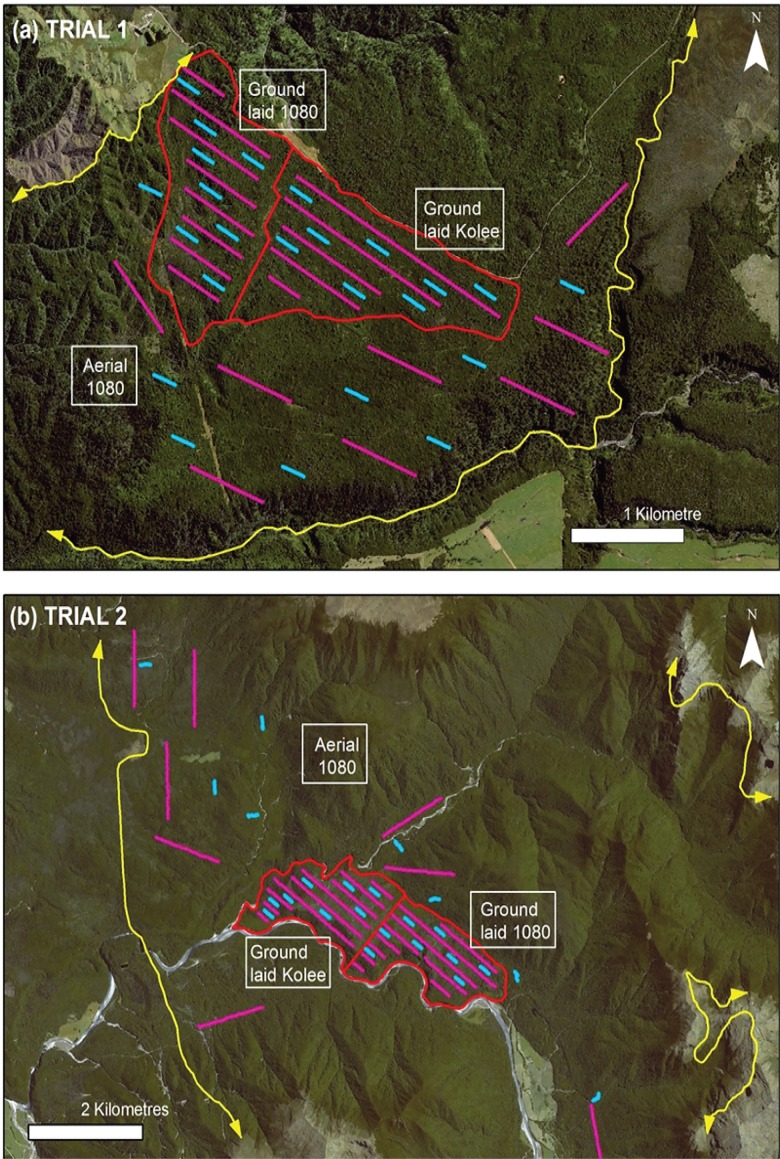
Satellite photographs (Airbus DS 2014) of the two trial sites with information overlaid to show the location of treatment blocks and monitoring lines. The ground baiting blocks are indicated by the red lines, and adjacent areas treated with aerial 1080 poisoning are indicated by yellow lines (arrows indicating continuation beyond the limits of the figure). Chewcard lines are shown in purple and trap lines in blue. The two images have different scales.

Pre-control possum monitoring during 8–12 June 2012 (Trial 1) and 24–29 July 2013 (Trial 2) used pre-baited chewcards (Connovation, Auckland, New Zealand). Between 173 and 206 cards were distributed throughout each block (S1 Table) at a spacing of 50m along GPS-specified lines that were separated by at least 200m (Fig 4). Chewcards were nailed to trees, about 10–20 cm above the ground. Because lines varied in length determined by the shape of the ground-baited blocks, the number of cards per line varied from 12 to 72 but as the aerial1080-baited areas were large, lines of a standard length were established in these areas, each with 25 chewcards. The locations of all cards were recorded as GPS waypoints, and all cards were collected after six nights for assessment by experienced staff. The procedure was repeated after control using the same lines, with cards being deployed during 16–19 August 2012 in Trial 1 about 5 weeks after control, and during 30 August-4 September 2013 in Trial 2 about 2 weeks after control.

The percentage population reduction given by each treatment was estimated as the mean percentage change in CCI calculated for each line. As the pre-control CCIs were greater than 90% for many of the lines, the CCI indices were likely to be ‘saturated’ (i.e. unable to change in linear proportion with changes in possum or rat density), which would tend to underestimate the possum and rat population reductions. The CCI data for each line before and after control were therefore arcsine-transformed to achieve a more linear index of population density (i.e. tCCI) [18]. Lines varied in length, giving differing numbers of cards per line, so the percentage change in tCCI was weighted by the number of cards on each line. The percentage change in tCCI was then used as the dependent variable in an ANOVA in the R statistical computing environment (version 2.15.1), and mean values were compared using Fisher’s LSD tests.

After chewcard monitoring was completed, a separate assessment of post-control possum abundance was made using the RTCI method [15]. In each block, 10 traps were set 20m apart for three nights along each of eight lines that were separated by at least 200m. Lines were located in between chewcard lines to estimate RTCI independently of the chewcard population indexing (Fig 4).

### Estimating costs

Costs (in New Zealand $) of control operations broadly comprise management costs and operational costs. Management costs are associated with planning control, obtaining the necessary permits, consulting and informing interested parties, and monitoring the effectiveness of operations. These costs are highly variable depending on the type and scale of an operation and have not been included in our comparison of the cost of the control methods we used. We based our comparison on the direct operational costs of bait and bait application, and the measured rate at which contractors applied baits. The comparison was restricted to the two treatments that used 1080 baits (aerial and ground-applied) since Kolee baits are not yet commercially available.

### Data accessibility

The raw data used in this study are publicly accessible on a website hosted by the parent organisation (Landcare Research Ltd) with a given URL address and a citable DOI (available at http://dx.doi.org/10.7931/J2JS9NCV or doi:dx.doi.org/10.7931/J2JS9NCV).

## Results

### Accuracy of navigation by contractors

**Fig 5:**
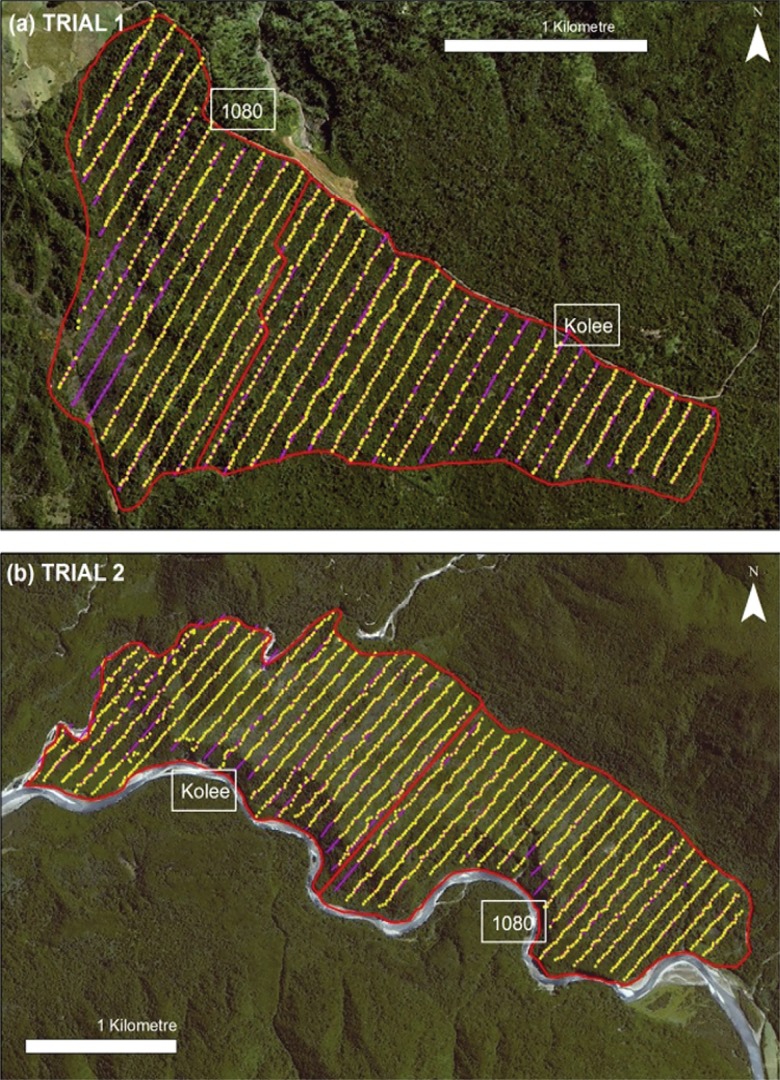
Satellite photographs (Airbus DS 2014) of the points where Kolee and 1080 baits were laid (yellow dots) by contractors following the GPS tracks of aerial prefeed lines (purple lines) in the two trials. The red line indicates the block boundaries. The two images have different scales.

Contractors accurately located and followed the GPS tracks recorded during aerial prefeeding. In Trial 1, toxic baits were hand laid along a total of 31.2km of prefeed tracks (Fig 5). Baiting points were either directly on the prefeed track or within a few metres of it, and all baiting points were well within the 60m prefeed swath centred on the track. Waterlogged ground prevented 1080 bait laying over approximately 9% of the prefeed lines, and Kolee bait laying over approximately 5%. Similarly, in Trial 2, contractors logged baiting points along 43.8 km of prefeed tracks, and baiting points were generally within a few metres of the tracks. Two exceptions were noted. First, in the western part of the Kolee block, contractors reported difficulty in obtaining repeatable GPS locations when at plots, leading to 12 waypoints being recorded incorrectly while, in fact, plots were probably located accurately. Secondly, the terrain in the gorge proved too difficult for some contractors to traverse while carrying loads of bait.

### Reduction of possum and rat populations – Trial 1

High CCIs, often approaching 100%, were recorded for both species (particularly possums) before toxic baiting, but interference was greatly reduced after control (S1 Table). For possums, CCIs fell to zero on most lines, with the exception of one CCI value on line 8 in the aerial 1080 block, where no reduction was observed: the rat CCI for that line fell from 79% to 4% confirming that 1080 baits had been distributed in the area.

**Fig 6:**
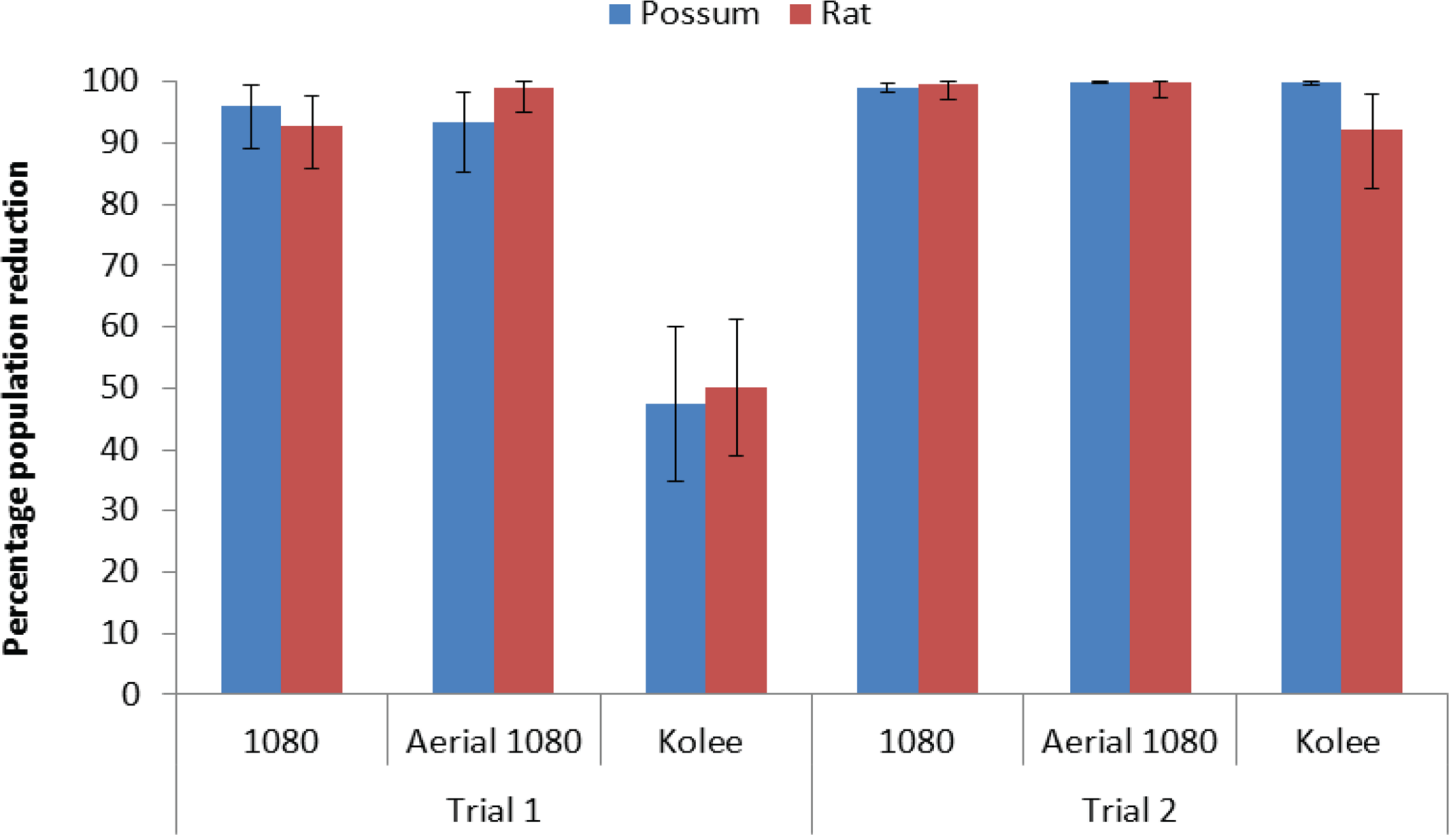
Mean percentage reduction in the line weighted, arcsine-transformed indices of Chewcard interference (tCCI) for possums and rats in the three treatment blocks of each trial, estimated from the change in 6 day chewcard interference. Means and SEs are back-transformed leading to asymmetrical SEs.

There was a highly significant overall difference in the reduction in the possum tCCI between blocks (F_2,19_ = 37.9, p < 0.001) (Fig 6). Ground-laid 1080 baits and aerial 1080 poisoning were similarly highly effective, with their mean reductions in tCCI (96.0% and 93.3%, respectively) significantly greater than the 47.4% reduction achieved by ground laid Kolee (p < 0.05 for both Fisher’s LSD tests). This difference was also reflected in the possum trapping results, with a more than nine-fold higher RTCI value in the block with ground laid Kolee (S2 Table).

Rat CCI reductions differed significantly between blocks (F_2,18_ = 9.6, p < 0.001) with aerial 1080 poisoning (98.9% reduction) and ground laid 1080 (92.8%) showing no significant difference in effectiveness, but both being more effective than ground laid Kolee (50.6%) (p < 0.05 for both Fisher’s LSD tests).

### Reduction of possum and rat populations – Trial 2

CCIs were zero on most lines for both possums and rats after control. Mean estimates of reduction in tCCI for both possums and rat populations were between 92.1% and 100% for all three treatments (Fig 6) with no statistical differences between treatments for either possums (F_2_,_2 1_ = 1.58, p = 0.23) or rats (F_2,18_ = 0.28, p = 0.76). Kolee baits were more effective in Trial 2, with reductions against possums and rats of 99.9% and 92.1% respectively being significantly greater than the reductions of 47.4% and 50.2% respectively in Trial 1 (p < 0.05 for both Fisher’s LSD tests).

No possums were trapped in any of the Trial 2 blocks (S2 Table) confirming the very high kills indicated by reduction in tCCI. This also shows the chewcard monitoring method used was more sensitive than the trapping method: over the two trials, chewcards detected possums survivors in four of the six treatments compared with two by trapping.

### Rate of coverage achieved by ground control contractors

Over the two trials, contractors took on average 1.67 min to complete each baiting plot and navigate to the next plot 20m away (Table 2). In the second trial, mean time per plot for Kolee bait laying was significantly greater than in the first trial (F_1,75_ = 4.22, p = 0.04), probably due to the doubling of the amount of Kolee laid. Expressed as a mean rate at which baiting lines were completed, this ranged from 0.72 km/h to 1.05 km/h, with the only significantly different result being the rate for Kolee, which was less in the second trial compared with the first (F_1,75_ = 5.77, p = 0.02). Overall, the mean rate of coverage was 0.88 km/h (SE = 0.03).

**Table 2:** Mean time spent moving to each bait plot, and speed of baiting.

Trial	Bait treatment	No. lines	No. plots	Mean (±SE) time per plot (min)	Mean (±SE) travel speed per line (km/h)
1	1080	13	610	1.85 (0.022)	0.85 (0.068)
	Kolee	23	704	1.42 (0.014)	1.05 (0.047)
2	1080	23	1067	1.69 (0.022)	0.86 (0.096)
	Kolee	20	1122	1.79 (0.015)	0.72 (0.042)
Combined		79	3503	1.67 (0.010)	0.88 (0.037)

### Comparisons of cost-effectiveness

**Fig 7:**
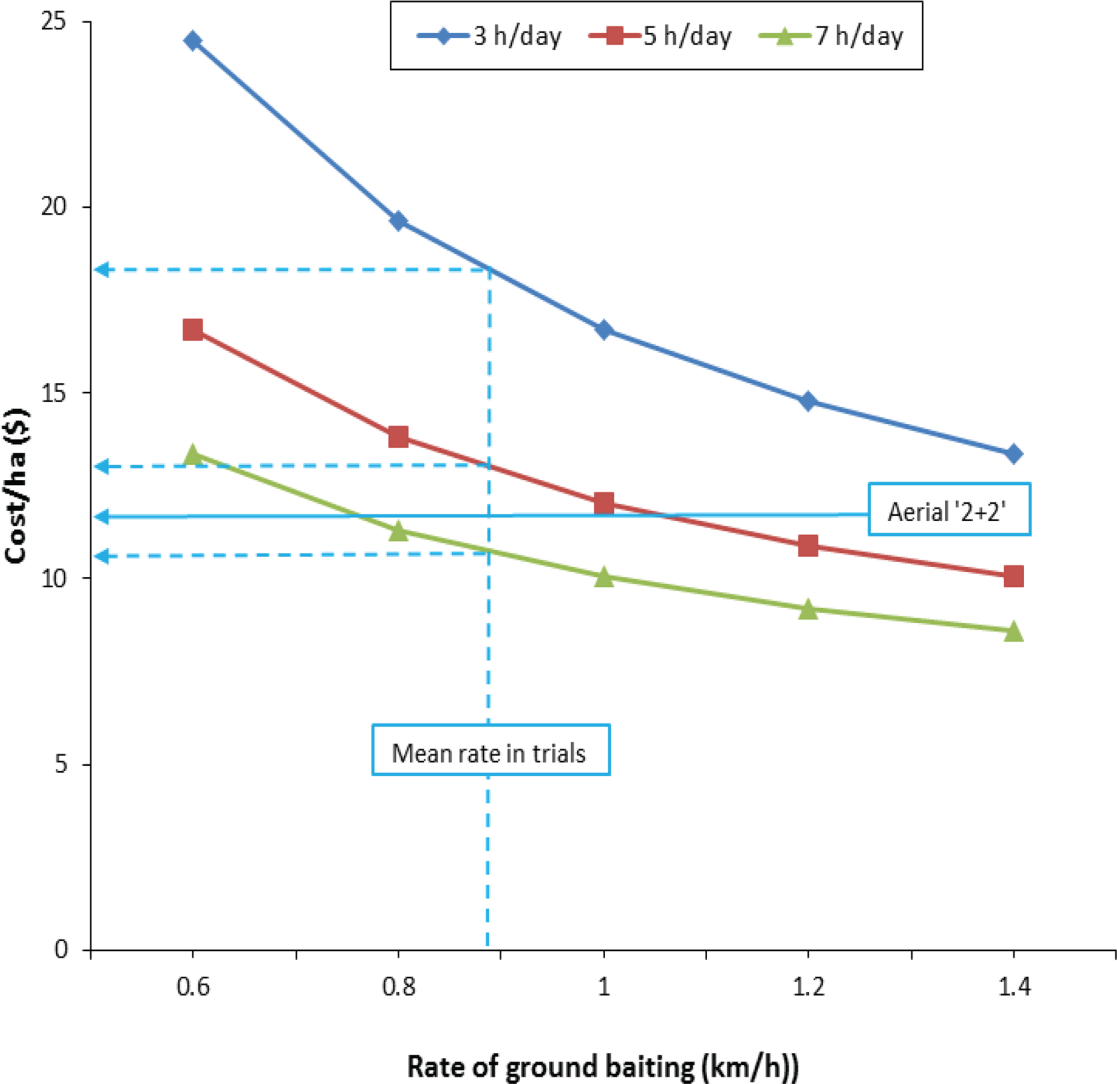
Predicted costs of baiting by aerial prefeed/ground control with 1080 baits, as used in this study. The horizontal axis shows the rate of ground baiting speed, which was between 0.71 and 1.05 km/h in this study. The dotted lines indicate the mean rate of baiting from the two trials, and the predicted costs of control under scenarios of 3, 5 and 7h of baiting per day. For comparison, the horizontal solid line indicates the cost ($NZ) of aerial 1080 baiting using a 180m flight-spacing, a mid-size helicopter (Squirrel AS350FS2), and a sowing bucket capable of carrying a maximum load of 500 kg of bait. The cost comprises only the cost of baits (prefeed and toxic bait both used at 2 kg/ha) and the cost of flying, including an estimated 10% extra for reloading. Other direct operational costs (including using deer repellent and positioning the helicopter at the operational site) and all operational management costs are excluded from the comparison.

In gently undulating terrain adjacent to road access, aerially prefed ground 1080 baiting was predicted to cost $11–18 per ha under the sowing specifications and mean recorded baiting speed for these trials, depending on the number of hours spent actually baiting each day (i.e. excluding the time spent travelling to and from lines) (Fig 7). For a 7h-long baiting day, the predicted costs were about 10% cheaper than a standard aerial broadcast 1080 baiting operation using 2 kg/ha prefeed and 2 kg/ha toxic bait for a similar outcome. Where greater distance is involved in travelling to and from operational sites, less time would be available for baiting lines. If baiting time was reduced to 5h per day, as is likely in winter or where greater travelling time is required, the method costs slightly more than aerial 1080 baiting, assuming the mean 0.88 km/h speed of bait laying. Under more extreme scenarios where 2h or more may be spent travelling to and from the baiting sites, leaving only as little as 3h available each day, the ground baiting method is predicted to cost about 50% more than aerial baiting, assuming a bait laying rate of 0.88 km/h. Increasing the rate of bait laying further improves cost effectiveness of the ground baiting method. For example, laying baits for 7h per day at a rate of 1.4 km/h would reduce the cost of control by about 30% compared with conventional aerial-broadcast poisoning: such a rate of progress may be achievable in, for example, pine plantations with little understorey vegetation on flat terrain.

**Fig 8:**
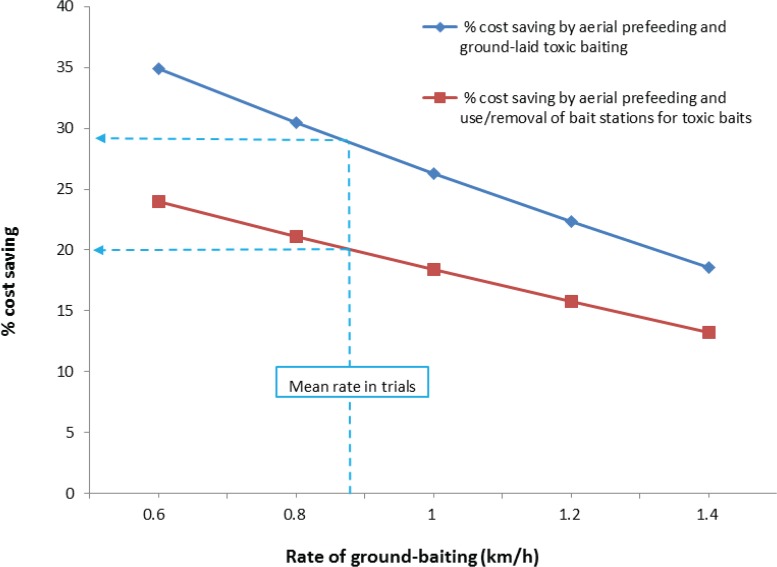
Predicted percentage cost-saving in ground-based control by prefeeding aerially rather than ground laying prefeed baits, and assuming 5h of bait laying per day. The blue line indicates savings when toxic baits are laid directly on the ground as in this study, while the red line estimates savings relative to a bait station operation in which an additional visit is necessary to remove baits stations. The dotted lines indicate the mean rate of baiting from the two trials, and the predicted cost-savings by prefeeding aerially. Greater proportional savings are obtained where the rate of ground baiting is slower (e.g. in difficult terrain), and where it is not necessary to deploy baits in bait stations.

The new method is also likely to be cheaper than traditional ground-based control methods (Fig 8). Relative to an operation in which prefeeding and poisoning are both done by ground-based contractors, the new method could reduce costs by about 28%, assuming 5h of bait laying per day and coverage at a rate of 0.88 km/h. If toxic baits must be presented in bait stations (i.e. weather-proof containers used for presenting baits), the saving gained through aerial prefeeding is reduced to around 20% because the overall cost is increased by an additional visit to remove bait stations.

**Fig 9:**
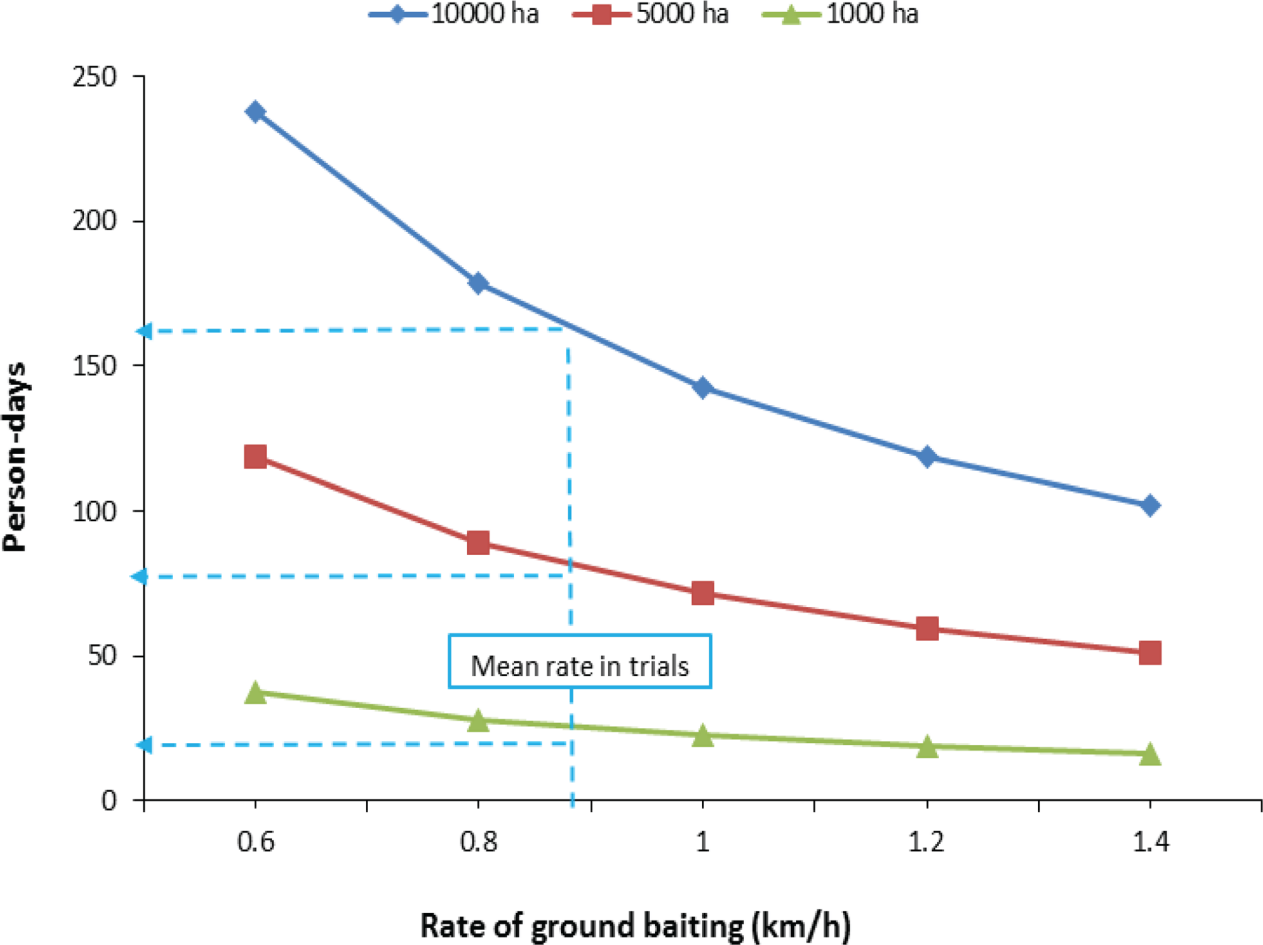
Predicted person-days required (based on 7h of baiting per day) for contractors to complete control, using the method tested here, in areas of 1000, 5000, and 10 000ha, at different baiting rates. The vertical dotted line indicates the mean rate of baiting from the two trials, and the horizontal dotted lines predict the person-days required to complete the three area sizes.

One disadvantage with the method may be the time taken to complete operations (Fig 9). At a baiting rate of 1 km/h, a contractor could be expected to treat 70ha per day if access was good enough to permit 7h of baiting per day. Assuming a contracting company used 10 staff with an average rate of baiting of 0.88 km/h, control using 1080 or Kolee pellets would be completed in blocks of 1000, 5000 and 10 000ha in 2.5, 8, and 16 days respectively. This assumes no delays due to bad weather. Clearly this is considerably slower than treatment by aerial poisoning, which can be expected to treat 5000ha or more per day with a single helicopter.

## Discussion

Two trials of a new specification-based approach to ground based control of possums have produced very encouraging results suggesting that, under favourable conditions of accessibility, topography and density of vegetation (as discussed below), very large reductions in possums and rat densities could be achievable at similar cost to conventional aerial 1080 baiting, and at lower cost compared with conventional ground based poison baiting. Compared to aerial 1080 baiting, our predictions of modest savings (under favourable conditions) in bait and bait applications costs are conservative as they do not include the greater compliance costs of aerial 1080 baiting compared with ground 1080 baiting. Indeed, in some instances, private landowner opposition to aerial 1080 may preclude use of that approach but not ground 1080 baiting, in which case the ground baiting technique is obviously far more ‘cost effective’. We have also shown in Trial 2 that another advantage over aerial 1080 baiting can be achieved by using cholecalciferol rather than 1080, since cholecalciferol is less likely to incur public opposition than 1080.

Compared to conventional ground based poisoning, larger cost savings are likely to accrue firstly because our approach of laying the baits on the ground avoids the need to use (and later remove) the bait stations typically used to hold bait (thereby incurring three rather than two visits in an operation where prefeeding occurs). Bait stations are bulky and time consuming to carry and install, but we made no allowance for these costs in our estimates of cost savings. The other major saving stems from using baits sparingly, by targeting their delivery to sites where they are most likely to be encountered by possums (unlike aerial application where baits cannot be placed at specific sites). The environmental risk of placing baits on the ground is considered to be negligible since exposure to baits is reduced through the restricted placement of baits in <5% of the operational area (including avoiding all watercourses and waterbodies), and operators are able to avoid distributing bait fragments, which are the main source of hazard to some birds and other small non-target animals [19].

In addition to the low cost of this method, long-run halving of the cost of ground based control of possums could also result from a reduction in the frequency of required control. The very high reductions in possums suggested as achievable by these trials are likely to exceed the reductions normally achieved in ground-based poisoning operations: while operations are normally assessed on the basis of residual population indices (e.g. trap catch or chewcard interference), actual reductions are thought to be generally in the region of around 80% (Dr P. Livingstone, TBfree New Zealand, pers. comm.) and further control would be ‘triggered’ sooner than for operations achieving near or complete elimination. For rats, however, the rate of population increase is not strongly related to the effectiveness of the control operation because recovery of rat populations is determined more by food availability than residual population density [20].

Contractors were clearly able to navigate prescribed routes very accurately. In addition to the skill of the personnel involved, this is also a reflection of the improved GPS units now available that are able to utilise satellite signals of any strength (not just those exceeding a threshold as was the case with earlier devices), generally providing continuous, accurate positioning data under a dense forest canopy. An exception was the inconsistent waypoint logging observed in the Kolee block of Trial 2. Since this occurred only on one part of the study site, within a period of 5h and affecting three GPS units, it is unlikely to have been caused by operator error. A possible explanation is the phenomenon of electromagnetic radiation from solar flares with consequent disturbance to the transmission of GPS signals from satellites to ground stations, generally lasting a few minutes [21] and possibly explaining the pulsed pattern of inaccurate logging along lines. Solar flares were common during 2013 as the sun reached the maximum of its 11 year cycle of solar activity, although no large flare was reported at the time of the inaccurate data logging [22].

There are some obvious constraints on the utility of this control method, including accessibility, topography and density of vegetation. In this initial investigation, we selected easily accessible trial sites where most of the terrain was easily traversed, but over most of the sites vegetation was very thick. While the rate of baiting is likely to be slower in heavily undulating terrain with gullies, the rate is likely to be more rapid where vegetation is less dense (i.e. in mature forest) than that encountered in this trial. The findings therefore suggest that the method is likely to be cost effective in many habitat types where access is good, but use of the technique will be limited by the proportion of an operational area that is too steep for contractors to undertake safely. While contractors were unable to lay baits in parts of the gorge area of Trial 2, monitoring lines did traverse several parts of the gorge and no possums or rats were detected or trapped. This suggests that a proportion of untreated steep terrain within a treated site may not jeopardise overall success. While the maximum tolerable dimensions of such excluded areas is as yet unknown (and no data are available on the movement of possums and rats specifically from areas of steep terrain to adjacent flatter habitat) the present study suggests that the baited plots had an effective ‘reach’ of up to at least 150m into steep terrain – this was the maximum distance between a baited plot and the most distant non baited part of the gorge.

Another constraint on this new control method may be the time taken to complete operations in areas where access may be restricted at certain times of the year. This may limit its use to smaller operational areas of 5000ha and less, where it would be feasible to complete the operation in a week or so. Furthermore, while the supply of bait to contractors was logistically straightforward in this small trial, it is likely to be complex in larger-scale operations and consequently to add additional cost. However, those areas for which an efficient alternative to aerial 1080 baiting is urgently required are those close to human settlement (e.g. water supply catchments). Such areas are likely to be more accessible (i.e. by roads and tracks, and farm/forest boundaries) than deep forest sites, and so efficient supply of baits to contractors is likely to be achievable. Use of GPS-specified bait ‘depots’ supplied by helicopter within or near the operational site may also prove practicable and efficient in less accessible sites.

The relatively poor control of both possums and rats achieved with Kolee in Trial 1 was unexpected given that the bait has been demonstrated to be highly palatable to both species (D. Morgan, unpubl. data), and a similar cereal-based cholecalciferol bait was effective in ‘mopping up’ both possums and rats that survived an initial control operation using cyanide paste bait [23]. The baiting pattern used clearly resulted in nearly all possums and rats finding and eating a lethal quantity of 1080 baits, but both species would have had to eat more Kolee bait than 1080 bait to ingest a lethal dose. Given the very high interference rate with chewcards before control at both sites, the population densities were greater than expected, and the application rate of Kolee bait was probably insufficient in Trial 1. When the rate was almost doubled in Trial 2, Kolee proved very effective in reducing populations of both possums and rats. Further testing at this higher rate is required for confirmation, but the need for an alternative to 1080 baits for controlling populations of possums and rats in areas close to human settlement could be met by the use of baits containing cholecalciferol.

A second possible explanation for the improvement in Kolee effectiveness in Trial 2 was the variation in the amount of cholecalciferol incorporated in the bait, i.e. 13% above specification in Trial 1 and 10% below specification in Trial 2. This suggests the possibility that the high concentration of cholecalciferol was aversive to some possums in Trial 1. However, this is considered an unlikely explanation as cholecalciferol assayed at 0.86% (i.e. 7.5% above specification) in a peanut paste, with no added masking flavour (such as cinnamon that was used in the present trials), proved highly effective in a cage trial, with the 10 possums tested consuming on average 37.2g of bait resulting in 100% mortality [24].

While the cost of cholecalciferol baits is likely to be higher than for 1080 baits due to the relatively high cost and lower toxicity compared with 1080, other research [24] has shown that if used at a low concentration (i.e. 0.1% wt:wt) in combination with a high concentration of inexpensive aspirin (i.e. 20%), cost could be halved with no loss of efficacy. Furthermore, in trials with captive possums, combination with aspirin results in a quicker (time to death = 12–24h) and more humane death (duration of toxicosis while conscious = 9–13h) compared with cholecalciferol alone (6d and 25h respectively). The combination of cholecalciferol and aspirin therefore appears a very promising alternative to 1080.

In summary, we have here reported promising results from a new ground-based approach to possum and rat control. Adoption of this approach by pest management agencies utilising pest control contractors would necessitate specification of the control method to be used rather than the traditional reliance on contractor’s preferred approaches [25]. Monitoring of contractor’s adherence to agreed protocols would be an essential part of operational monitoring and we suggest that this should form the basis for control contracts rather than attainment of specified pest population indices (e.g. RTCI). Population monitoring should still be undertaken with the new method, but rather than being used to assess contractor performance, it should be used primarily to determine the limitations on the use of the new method.

## Supporting Information

S1 Table Interference with chewcards before and after control by possums and rats in Trials 1 and 2. (DOCX)

S2 Table Summary of the residual (post-control) possum Trap Catch index values (± 95% CI) in the three treatment blocks in each of the two trial sites. (DOCX)
