# Supplementary material for: Aerial Prefeeding Followed by Ground Based Toxic Baiting for More Efficient and Acceptable Poisoning of Invasive Small Mammalian Pests
Source: PLoS One. 2015 Jul 28;10(7):e0134032. doi: 10.1371/journal.pone.0134032 (PMC4517755; doi:10.1371/journal.pone.0134032)
Supplement: S1 Table — (DOCX) [file pone.0134032.s001.docx]

**S1 Table – Interference with chewcards before and after control by possums and rats in Trials 1 and 2.**

| **Trial** | **Treatment** | **Line** | **No. of chewcards** | **% Possum interference** | | **% Rat interference** | |
| --- | --- | --- | --- | --- | --- | --- | --- |
|  |  |  |  | **Before control** | **After control** | **Before control** | **After control** |
| 1 | Aerial 1080 | 1 | 24 | 71 | 0 | 29 | 0 |
| 1 | Aerial 1080 | 2 | 24 | 96 | 4 | 33 | 0 |
| 1 | Aerial 1080 | 3 | 24 | 79 | 0 | 38 | 0 |
| 1 | Aerial 1080 | 4 | 24 | 100 | 0 | 46 | 0 |
| 1 | Aerial 1080 | 5 | 24 | 100 | 0 | 21 | 0 |
| 1 | Aerial 1080 | 6 | 24 | 92 | 8 | 88 | 29 |
| 1 | Aerial 1080 | 7 | 24 | 88 | 0 | 63 | 0 |
| 1 | Aerial 1080 | 8 | 24 | 42 | 48 | 79 | 4 |
| 1 | Ground-laid 1080 | 1 | 15 | 93 | 0 | 87 | 60 |
| 1 | Ground-laid 1080 | 2 | 35 | 91 | 6 | 77 | 3 |
| 1 | Ground-laid 1080 | 3 | 32 | 94 | 28 | 63 | 16 |
| 1 | Ground-laid 1080 | 4 | 25 | 100 | 4 | 44 | 12 |
| 1 | Ground-laid 1080 | 5 | 25 | 92 | 0 | 8 | 0 |
| 1 | Ground-laid 1080 | 6 | 22 | 91 | 0 | 14 | 0 |
| 1 | Ground-laid 1080 | 7 | 22 | 59 | 0 | 9 | 0 |
| 1 | Ground-laid 1080 | 8 | 18 | 82 | 11 | 29 | 0 |
| 1 | Kolee | 1 | 72 | 75 | 32 | 97 | 67 |
| 1 | Kolee | 2 | 55 | 89 | 49 | 53 | 40 |
| 1 | Kolee | 3 | 35 | 100 | 46 | 37 | 11 |
| 1 | Kolee | 4 | 32 | 91 | 66 | 22 | 3 |
| 1 | Kolee | 5 | 12 | 100 | 67 | 25 | 0 |
| 2 | Aerial 1080 | 1 | 25 | 48 | 0 | 40 | 0 |
| 2 | Aerial 1080 | 2 | 25 | 80 | 0 | 80 | 0 |
| 2 | Aerial 1080 | 3 | 25 | 92 | 0 | 20 | 0 |
| 2 | Aerial 1080 | 4 | 25 | 88 | 0 | 76 | 0 |
| 2 | Aerial 1080 | 5 | 25 | 92 | 0 | 24 | 0 |
| 2 | Aerial 1080 | 6 | 25 | 80 | 0 | 68 | 0 |
| 2 | Aerial 1080 | 7 | 25 | 87 | 0 | 70 | 8 |
| 2 | Aerial 1080 | 8 | 25 | 88 | 0 | 48 | 0 |
| 2 | Ground-laid 1080 | 1 | 23 | 75 | 0 | 35 | 0 |
| 2 | Ground-laid 1080 | 2 | 34 | 62 | 9 | 44 | 0 |
| 2 | Ground-laid 1080 | 3 | 23 | 78 | 0 | 26 | 0 |
| 2 | Ground-laid 1080 | 4 | 23 | 61 | 0 | 17 | 0 |
| 2 | Ground-laid 1080 | 5 | 23 | 65 | 0 | 22 | 0 |
| 2 | Ground-laid 1080 | 6 | 23 | 77 | 0 | 14 | 0 |
| 2 | Ground-laid 1080 | 7 | 23 | 100 | 0 | 30 | 4 |
| 2 | Ground-laid 1080 | 8 | 13 | 100 | 8 | 64 | 0 |
| 2 | Kolee | 1 | 23 | 77 | 0 | 0 | 0 |
| 2 | Kolee | 2 | 12 | 67 | 0 | 25 | 0 |
| 2 | Kolee | 3 | 23 | 61 | 0 | 4 | 13 |
| 2 | Kolee | 4 | 23 | 70 | 0 | 4 | 0 |
| 2 | Kolee | 5 | 23 | 70 | 0 | 0 | 0 |
| 2 | Kolee | 6 | 23 | 96 | 4 | 22 | 0 |
| 2 | Kolee | 7 | 23 | 96 | 0 | 22 | 0 |
| 2 | Kolee | 8 | 23 | 91 | 0 | 13 | 0 |
