# Supplementary material for: Aerial Prefeeding Followed by Ground Based Toxic Baiting for More Efficient and Acceptable Poisoning of Invasive Small Mammalian Pests
Source: PLoS One. 2015 Jul 28;10(7):e0134032. doi: 10.1371/journal.pone.0134032 (PMC4517755; doi:10.1371/journal.pone.0134032)
Supplement: S2 Table — (DOCX) [file pone.0134032.s002.docx]

**S2 Table - Summary of the residual (post-control) possum Trap Catch index values (± 95% CI) in the three treatment blocks in each of the two trial sites**

| **Bait treatment** | **Trial 1** | **Trial 2** |
| --- | --- | --- |
| Ground-laid 1080 | 0 | 0 |
| Ground-laid Kolee | 3.8 (3.1) | 0 |
| Aerial 1080 | 0.4 (1.0) | 0 |
